# Smoking cessation decreases arterial blood pressure in hypertensive smokers: A subgroup analysis of the randomized controlled trial GENTSMOKING

**DOI:** 10.18332/tid/186853

**Published:** 2024-05-16

**Authors:** Patricia V. Gaya, Guilherme Wesley P. Fonseca, Lucas Tsuyoshi Tanji, Tania O. Abe, Maria Janieire N. N. Alves, Paulo Caleb Junior de Lima Santos, Fernanda M. Consolim Colombo, Jaqueline R. Scholz

**Affiliations:** 1 Programa de Prevenção, Instituto do Coração, Hospital das Clinicas (HCFMUSP), Faculdade de Medicina, Universidade de São Paulo, São Paulo, Brazil; 2 Escola de Educação Física e Esporte, Universidade de São Paulo, Brazil; 3 Faculdade de Medicina (FMUSP), Universidade de Sao Paulo, São Paulo, Brazil; 4 Instituto do Coração, Hospital das Clinicas (HCFMUSP), Faculdade de Medicina, Universidade de São Paulo, São Paulo, Brazil; 5 Departamento de Farmacologia, Escola Paulista de Medicina, Universidade de São Paulo, São Paulo, Brazil; 6 Unidade de Hipertensão, Instituto do Coração, Hospital das Clinicas (HCFMUSP), Faculdade de Medicina, Universidade de São Paulo, São Paulo, Brazil

**Keywords:** blood pressure, smoking cessation, hypertension management

## Abstract

**INTRODUCTION:**

High blood pressure in hypertensive smokers is affected by nicotine consumption. This study aimed to evaluate the effect of smoking cessation treatments on blood pressure in hypertensive smokers.

**METHODS:**

A total of 113 hypertensive smokers on antihypertensives during smoking cessation treatment in the randomized controlled trial GENTSMOKING were considered for analysis. At Baseline (T0) and Week 12 (T12), systolic and diastolic blood pressure (SBP and DBP), and heart rate (HR) were measured using a semi-automated digital oscillometric device. Mean arterial pressure (MAP) and delta differences for SBP, DBP, HR, and MAP were calculated. Smoking cessation was confirmed by measuring carbon monoxide (CO) in exhaled air.

**RESULTS:**

After 12 weeks of treatment, 72 participants ceased smoking (cessation group) and 41 did not (no cessation group). At T0, there was no statistically meaningful difference between groups with respect to age, body mass index, CO, and daily cigarette consumption. At T12, daily cigarette consumption and CO had decreased in both groups (p<0.001). The cessation group showed decreased SBP (131 ± 2 vs 125 ± 2 mmHg, p=0.004), DBP (79 ± 1 vs 77 ± 1 mmHg, p=0.031), MAP (96 ± 1 vs 93 ± 1 mmHg, p=0.005), and HR (79 ± 1 vs 74 ± 1 beats/min, p=0.001), and increased body weight (77.4 ± 2.1 vs 79.2 ± 2.2 kg, p<0.001). No significant differences were seen for these variables in the no cessation group. Decrease in blood pressure was significantly higher among hypertensive participants with SBP ≥130 mmHg: SBP (145 ± 2 vs 132 ± 2 mmHg, p<0.001), DBP (85 ± 2 vs 80 ± 1 mmHg, p=0.002), MAP (105 ± 1 vs 97 ± 1 mmHg, p<0.001), and HR (81 ± 2 vs 74 ± 2 beats/min, p=0.002). A positive correlation was found between HR and CO (r=0.34; p=0.001).

**CONCLUSIONS:**

Smoking cessation treatment reduced blood pressure in hypertensive smokers, allowing them to reach therapeutic targets for hypertension management. Smoking cessation has a positive impact on hypertension treatment; therefore, it should be encouraged in clinical practice.

**CLINICALTRIALS.GOV IDENTIFIER:**

NCT03362099

## INTRODUCTION

Smoking, hypertension, dyslipidemia, and diabetes mellitus are main risk factors for cardiovascular disease^1^. Smoking cessation is the most effective lifestyle intervention for prevention of cardiovascular and neoplastic diseases, as well as chronic obstructive pulmonary disease, and smoking cessation treatments improve the control of autoimmune inflammatory diseases^2^. Impaired endothelial function, arterial stiffness, increased inflammation, lipid alteration, and prothrombotic factors are associated with smoking and appear to initiate as well as accelerate the atherothrombotic process, eventually leading to cardiovascular events^3^.

Regarding blood pressure (BP), a 10-mmHg reduction in systolic blood pressure (SBP) has been associated with a 10% decrease in cardiovascular events and a 36% decrease in strokes^4^. Current guidelines for hypertension management have established BP targets <130 and 80 mmHg for SBP and diastolic blood pressure (DBP), respectively^5^. Although pharmacological strategies are usually implemented to help achieve hypertension control, the impact of smoking cessation on hemodynamic parameters as a clinical strategy to achieve BP targets in smokers has rarely been discussed or even considered.

Smoking exerts an acute hypertensive effect via stimulation of the sympathetic nervous system^6,7^. Hypertensive smokers are more likely to develop severe forms of hypertension^8^, including renal dysfunction^9,10^ and left ventricular hypertrophy^11^. However, available data on chronic smoking do not allow the establishment of a clear relationship between smoking and hypertension, based on studies showing that hypertension prevalence was no higher among smokers compared to the general population^12^. Other studies have reported that smoking cessation was significantly associated with an increased risk of hypertension^13^, and that current smoking was not a risk factor for hypertension^14,15^.

In the smoking cessation program developed at the outpatient clinic of Instituto do Coração [Heart Institute] (InCor) of Hospital das Clínicas [University Hospital] of Faculdade de Medicina [Faculty of Medicine] of Universidade de São Paulo [University of São Paulo] (HCFMUSP), one can often find patients with reduced BP after smoking cessation. Nevertheless, there are limited data systematically assessing such association, considering that hypertensive smokers undergoing hypertension treatment are rarely subjected to simultaneous smoking cessation treatment by cardiologists. This simplified approach restricted smoking cessation guidance to counseling alone, with reduced effectiveness. It is not feasible for a clinical team to measure the real impact of smoking cessation on BP levels in hypertensive smokers; these can be observed only in smoking treatment programs with high cessation efficacy.

Therefore, the aim of this work, consisting of a subgroup analysis of participants from the randomized controlled trial GENTSMOKING (ClinicalTrials.gov Identifier: NCT03362099) involving 361 smokers admitted to smoking treatment with varenicline, bupropion, or their combination, was to evaluate the efficacy of smoking cessation treatment in lowering BP levels of 113 hypertensive smokers.

## METHODS

### Study design and population

GENTSMOKING was conducted at a single center, InCor/HCFMUSP (São Paulo, Brazil). From November 2017 to March 2022, the study prospectively enrolled 361 smokers who sought smoking cessation treatment at a specialized center, smoked at least five cigarettes per day in the previous year, were aged 18–79 years, male or female, with sedentary lifestyle (no regular physical activities). Exclusion criteria included: alcohol or drug users; women of childbearing potential; subjects with significant clinical hepatic, renal, or gastrointestinal disorders; those who had unstable cardiovascular diseases or psychiatric disorders; those who had seizures or were at risk of developing them; those who suffered from head trauma or brain tumor; those who had previous allergic reactions to bupropion or varenicline; as well as those who did not meet protocol requirements.

Antitobacco drugs considered for this analysis were bupropion and varenicline, administered alone or in combination, during a treatment protocol of 12 weeks. A total of 113 participants were hypertensive on treatment with antihypertensives; drug class and dosage were not modified during smoking cessation treatment. Almost 45% of participants made use of only one antihypertensive drug, 40.5% used two drugs, 11% used three drugs, and 3.5% used four drugs; antihypertensives included angiotensin receptor blockers (63%), beta blockers (34%), diuretics (27%), calcium channel antagonists (25%), and converting enzyme inhibitors (23%). Smoking status was collected through self-reporting and confirmed using carbon monoxide (CO) in exhaled air, at all visits. Smoking cessation treatment was considered successful when participants were continuously abstinent from smoking between Weeks 8 and 12. The Issa situational smoking score and Fagerström test for nicotine dependence score were also collected.

### Assessment of carbon monoxide

Bedfont Smokerlyzer^®^ (Bedfont^®^ Scientific Ltd., UK) was used to measure carbon monoxide concentration in exhaled air. Participants were instructed to inhale deeply and hold their breath for a pre-set amount of time (15 s). After three beeping sounds, participants were told to exhale the air trapped in their lungs slowly into the mouthpiece of the CO-measuring device until their lungs were completely empty. Results are shown on the device screen and expressed as ppm and % equivalent to carboxyhemoglobin, a compound formed in the blood when carbon monoxide occupies the positions on the hemoglobin molecule normally taken by oxygen. The cessation group comprised participants who reported continuous abstinence between Weeks 8 and 12, confirmed through carbon monoxide concentration (cutoff ≤3 ppm). Such cutoff values are only obtained with at least 72 hours of total abstention from smoking^16^.

### Assessment of hemodynamic parameters

SBP, DBP, mean arterial pressure (MAP), and heart rate (HR) were assessed in the mornings for 10 min with the participant resting in a seated position in a quiet place, using a validated semi-automated monitor (Omron HEM-9210T, USA) always in the same arm. Measurement was repeated twice (within 2 min of each other) when SBP >140 mmHg or DBP >90 mmHg; the second measurement was considered as reference^17^.

SBP, DBP, HR, body weight (BW), and CO were evaluated at all study visits. MAP was calculated using the formula: ([2 × DBP] + SBP)/3. Delta differences (Δ) were calculated for SBP, DBP, HR, and MAP by subtracting baseline values from results at Week 12. All variables were recorded at three distinct time points: before any intervention at Baseline (T0), at Week 4 (T4), and at Week 12 (T12).

### Statistical analysis

Data are presented as mean ± standard deviation (SD), median, and 95% confidence interval (95% CI), or as frequency and percentage. The one-sample Kolmogorov-Smirnov test was used to assess the normal distribution of variables. Repeated measures analysis of variance (two-way ANOVA) was used to compare hemodynamic and smoking variables at different time points between groups (no cessation vs cessation). The differences between post-intervention (T12) and baseline (T0) values were calculated and expressed as delta (Δ). The Mann-Whitney U test was used to compare calculated deltas for all hemodynamic variables, and the chi-square test was used to compare categorical data, such as medication and comorbidity distribution. Spearman’s correlation was used to determine the association between hemodynamic and smoking variables. Linear mixed model analysis was performed for changes between Weeks 0 and 12 in SBP, DBP, MAP, and HR adjusted for confounding variables, such as BW, age, sex, and use of anti-hypertensive drugs.

The Statistical Package for the Social Sciences version 23 (SPSS Inc., USA) was used to perform all statistical analyses. Significant was set at p<0.05.

## RESULTS

One hundred and thirteen hypertensive smokers in GENTSMOKING were divided into two groups (no cessation, n=41; cessation, n=72) according to their smoking status from Week 8 to Week 12 of treatment (Table 1). At T0, there were no differences in age (58 ± 8 vs 58 ± 10 years), BW (74.8 ± 14.5 vs 77.4 ± 18.9 kg, p=0.46), height (1.65 ± 0.08 vs 1.64 ± 0.10 m, p=0.68), body mass index (BMI) (27.3 ± 4.2 vs 28.2 ± 5.5 kg/m^2^, p=0.35), and CO (12.9 ± 4.5 vs 12.1 ± 4.8 ppm, p=0.43) between participants in the no cessation and cessation groups, respectively. Nicotine dependence as per Issa situational smoking score (3.1 ± 0.6 vs 3.2 ± 0.5, p=0.95) and Fagerström score (7.5 ± 1.6 vs 7.2 ± 1.8, p=0.33) was statistically the same in the no cessation and cessation groups, respectively. Most participants in the no cessation and cessation groups were White (83 vs 82%, p=0.92) and female (59 vs 56%, p=0.84), respectively. Distribution of comorbidities and associated diseases, including diabetes mellitus (20 vs 19%, p=1.00), coronary artery disease (12 vs 13%, p=1.00), dyslipidemia (44 vs 38%, p=0.55), chronic obstructive pulmonary disease (20 vs 14%, p=0.44), and other diseases (24 vs 29%, p=0.66), as well as the number of antihypertensives used (1.7 vs 1.7 drugs, p=0.9) were not statistically different between participants in the no cessation and cessation groups, respectively (Table 1).

**Table 1:** Demographics and clinical characteristics of participants by smoking status

*Characteristics*	*No Cessation (N=41) n (%)*	*Cessation (N=72) n (%)*	*p*
Age (years), mean ± SD	58 ± 8	58 ± 10	0.93
Female	24 (59)	40 (56)	0.84
White	34 (83)	59 (82)	0.92
Body weight (kg), mean ± SD	74.8 ± 14.5	77.4 ± 18.9	0.46
Height (m), mean ± SD	1.65 ± 0.08	1.64 ± 0.10	0.68
Body mass index (kg/m2), mean ± SD	27.3 ± 4.2	28.2 ± 5.5	0.35
Diabetes mellitus	8 (20)	14 (19)	1.00
Coronary artery disease	5 (12)	9 (13)	1.00
Depression	7 (17)	16 (22)	0.63
Anxiety	12 (29)	16 (22)	0.50
Dyslipidemia	18 (44)	27 (38)	0.55
Chronic obstructive pulmonary disease	8 (20)	10 (14)	0.44
Other diseases	10 (24)	21 (29)	0.66
Medications (n), mean ± SD	3.5 ± 1.6	3.3 ± 1.5	0.46
Antidepressive agents	20 (49)	29 (40)	0.84
Antihypertensive medications (n), mean ± SD	1.7 ± 0.8	1.7 ± 0.6	0.90
Fagerström score, mean ± SD	7.5 ± 1.6	7.2 ± 1.8	0.33
Issa score, mean ± SD	3.1 ± 0.6	3.2 ± 0.5	0.95
Cigarettes/day, mean ± SD	23.1 ± 10.4	19.8 ± 8.1	0.06
Carbon monoxide (ppm), mean ± SD	12.9 ± 4.5	12.1 ± 4.8	0.43

**Figure 1:**
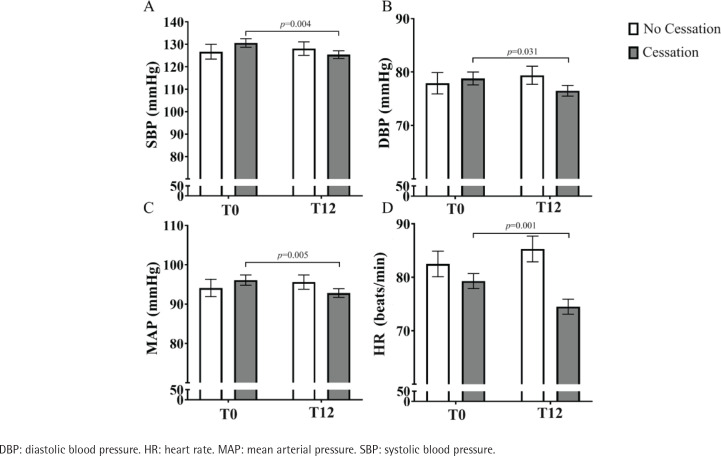
Hemodynamic parameters at the beginning (T0) and end of treatment (T12) for participants in the cessation and no cessation groups

When comparing hemodynamic parameters using repeated measures from T0 to T12 between both groups (Figure 1), the cessation group showed decreased SBP (131 ± 2 vs 125 ± 2 mmHg, p=0.004, Panel A), DBP (79 ± 1 vs 77 ± 1 mmHg, p=0.031, Panel B), MAP (96 ± 1 vs 93 ± 1 mmHg, p=0.005, Panel C), and HR (79 ± 1 vs 74 ± 1 beats/min, p=0.001, Panel D), and increased BW (77.4 ± 2.1 vs 79.2 ± 2.2 kg, p<0.001). No differences in these hemodynamic parameters were observed in the no cessation group. The following values were established by comparing mean delta differences between the cessation and no cessation groups: ΔSBP (-6 [-15 – 6] vs 5 [-10 – 11] mmHg, p=0.041); ΔDBP (-2 [-8 – 4] vs 2 [0 – 7] mmHg, p=0.036); ΔMAP (-3 [-9 – 6] vs 4 [-4 – 8] mmHg, p=0.039); and ΔHR (-4 [-14 – 5] vs 1 [-5 – 5] beats/min, p =0.020).

**Figure 2:**
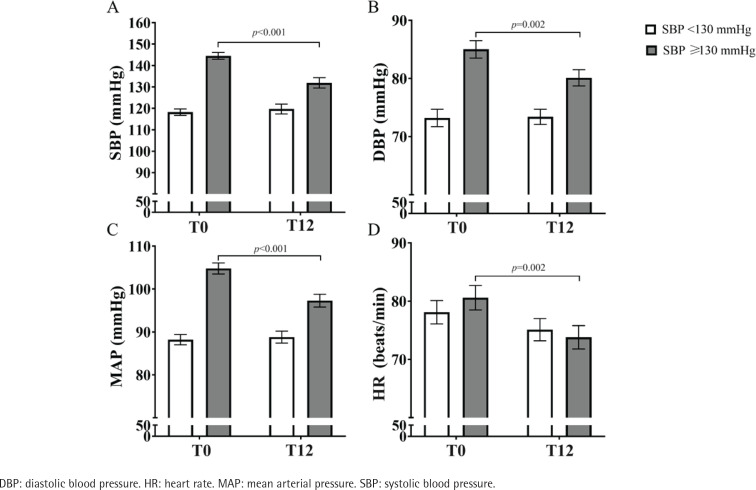
Hemodynamic parameters at the beginning (T0) and end of treatment (T12) for participants in the cessation group with SBP <130 mmHg and SBP ≥130 mmHg

To determine whether initial BP and smoking cessation were correlated, the cessation group (n=72) was divided into two groups: participants with SBP <130 mmHg (n=38) and participants with SBP ≥130 mmHg (n=34) (Figure 2). Comparing hemodynamic parameters from T0 to T12 in participants with SBP ≥130 mmHg led to decreased SBP (145 ± 2 vs 132 ± 2 mmHg, p<0.001, Panel A), DBP (85 ± 2 vs 80 ± 1 mmHg, p=0.002, Panel B), MAP (105 ± 1 vs 97 ± 1 mmHg, p<0.001, Panel C), and HR (81 ± 2 vs 74 ± 2 beats/min, p=0.002, Panel D). Comparisons of mean delta differences between SBP ≥130 mmHg and SBP <130 mmHg showed a greater decrease in ΔSBP (-15 [-20 – -3] vs 1 [-7 – 10] mmHg, p<0.001), ΔDBP (-5 [-10 – 2] vs 0 [-7 – 8] mmHg, p=0.023), and ΔMAP (-8 [-13 – -2] vs 0 [-5 – 7] mmHg, p<0.001) in participants with higher SBP values, whereas no difference in ΔHR (-5 [-15 – -1] vs -3 [-13 – 7] mmHg, p=0.196) was noted between groups.

With regard to pharmacological treatment at T0 and T12 in the cessation group, only varenicline users showed decreased SBP (130 ± 3 vs 122 ± 2 mmHg, p=0.001), DBP (79 ± 2 vs 75 ± 1 mmHg, p=0.007), and MAP (96 ± 2 vs 91 ± 1 mmHg, p=0.001), whereas HR values tended to decrease (79 ± 2 vs 76 ± 2 beats/min, p=0.065). Furthermore, there were no differences in hemodynamic parameters from T0 to T12 for varenicline or bupropion, as well as their combination, for participants in the no cessation group (Table 2).

**Table 2:** Hemodynamic variables by smoking status groups and smoking cessation treatment at the beginning (T0), middle (T4), and end of treatment (T12)

*Group*	*Variables*	*Medication*	*Baseline (T0) Mean ± SD*	*Week 4 (T4) Mean ± SD*	*Week 12 (T12) Mean ± SD*
**Cessation**	SBP (mmHg)	Varenicline (n=42)	130 ± 3	125 ± 2*	122 ± 2*
		Bupropion (n=3)	132 ± 9	133 ± 4	129 ± 9
		Combination (n=27)	132 ± 3	132 ± 4	130 ± 3
	DBP (mmHg)	Varenicline (n=42)	79 ± 2	76 ± 2*	75 ± 1*
		Bupropion (n=3)	81 ± 6	81 ± 3	82 ± 5
		Combination (n=27)	78 ± 2	78 ± 5	78 ± 2
	MAP (mmHg)	Varenicline (n=42)	96 ± 2	92 ± 2*	91 ± 1*
		Bupropion (n=3)	98 ± 7	98 ± 3	98 ± 5
		Combination (n=27)	96 ± 2	97 ± 4	95 ± 2
	HR (beats/min)	Varenicline (n=42)	79 ± 2	76 ± 2*	76 ± 2*
		Bupropion (n=3)	87 ± 7	80 ± 4	78 ± 7
		Combination (n=27)	78 ± 2	74 ± 3	72 ± 2*
**No Cessation**	SBP (mmHg)	Varenicline (n=3)	130 ± 16	127 ± 7	120 ± 11
		Bupropion (n=4)	144 ± 8	142 ± 5	141 ± 5
		Combination (n=19)	123 ± 4	124 ± 3	126 ± 3
	DBP (mmHg)	Varenicline (n=3)	80 ± 6	79 ± 4	80 ± 7
		Bupropion (n=4)	87 ± 3	80 ± 2	79 ± 3
		Combination (n=19)	76 ± 1	78 ± 2	79 ± 2
	MAP (mmHg)	Varenicline (n=3)	97 ± 7	96 ± 4	93 ± 7
		Bupropion (n=4)	106 ± 4	97 ± 3	100 ± 3
		Combination (n=19)	92 ± 2	94 ± 2	95 ± 2
	HR (beats/min)	Varenicline (n=3)	80 ± 10	81 ± 5	78 ± 13
		Bupropion (n=4)	93 ± 5	92 ± 4	90 ± 7
		Combination (n=19)	81 ± 2	82 ± 2	85 ± 3

SBP: systolic blood pressure. DBP: diastolic blood pressure. MAP: mean arterial pressure. HR: heart rate.

* Two-way ANOVA was used, statistically significant difference compared to T0 (p<0.05).

**Figure 3:**
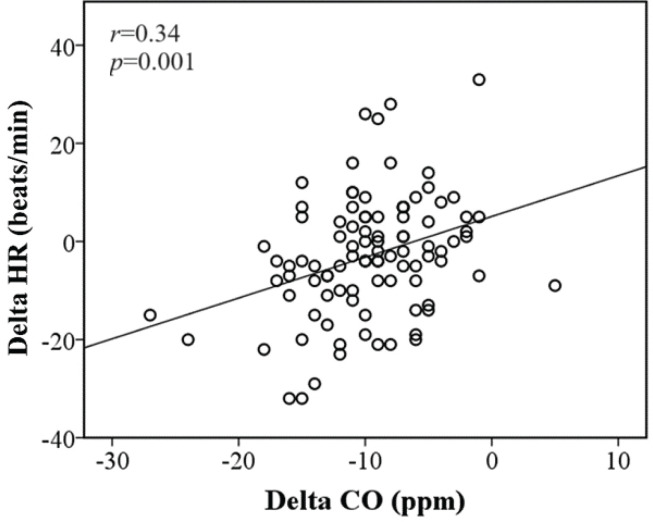
Spearman’s correlation for delta differences in HR (heart rate) and CO (carbon monoxide) in 113 participants

Cigarettes smoked per day and CO concentration in exhaled air were reduced between T0 and T12 in both groups (p<0.0001). In addition, there was a moderately positive correlation between delta differences in HR and CO (r=0.34; p=0.001) (Figure 3).

Linear mixed model analysis was used to assess changes in hemodynamic variables as dependent variables adjusted by age, sex, ΔBW, ΔBMI, Δ cigarette consumption, cessation status, and presence of coronary artery disease, chronic obstructive pulmonary disease, and diabetes mellitus. Statistical association was found for ΔSBP (F=4.247; p=0.042, Supplementary file Table 1), ΔMAP (F=4.684; p=0.033, Supplementary file Table 3), and ΔHR (F=5.821; p=0.018, Supplementary file Table 4), while cessation showed a tendency to be associated with ΔDBP (F=3.594; p=0.068, Supplementary file Table 2).

## DISCUSSION

The impact of smoking cessation on BP in hypertensive smokers is noteworthy; however, few studies have compared data from the same participants before and after smoking cessation treatment. An example is the retrospective cohort study conducted by Tsai et al.^18^, in which data were collected from participants of a smoking cessation program in an outpatient clinic of a tertiary medical center in Taiwan from 2017 to 2018. This study investigated the effects of smoking cessation on BP control in hypertensive smokers after 12 weeks of treatment. A significant decrease was found in SBP (-5.4 mmHg) and DBP (-3.6 mmHg) in participants who quit smoking. In our study, reductions in SBP (-6 mmHg) and DBP (-2 mmHg) in hypertensive smokers who ceased smoking after 12 weeks of treatment were observed. This reduction was even more pronounced in participants with higher BP levels, leading to reductions in SBP and DBP of -13 mmHg and -5 mmHg, respectively, amongst uncontrolled hypertensive smokers (SBP >130 mmHg). This finding highlights the significant impact of smoking cessation on hypertensive smokers with inadequate BP control.

Our study shows that participants with hypertension who underwent treatment and ceased smoking experienced a significant decrease in SBP, DBP, HR, and MAP, even though no additional optimization of antihypertensive treatment was required. This suggests that smoking cessation alone can improve hemodynamic parameters in participants with hypertension. Participants with higher BP showed the greatest decrease in BP.

Oncken et al.^19^ conducted a study to assess clinical and ambulatory BP and HR. Twenty-four-hour urinary catecholamine concentrations were obtained at baseline and again at Week 6 to assess the impact of smoking cessation on BP control in 66 hypertensive women. They found a significant reduction of 3.6 mmHg in SBP and HR decreased by 7 beats/min in 19 women who had ceased smoking. In addition, norepinephrine and epinephrine levels were reduced in this group.

In fact, in a large prospective cohort of 28236 women in the Women’s Health Study^20^, cigarette smoking was modestly associated with an increased risk of developing hypertension, and the effect was strongest among women smoking at least 15 cigarettes per day.

In the Guizhou Population Health Cohort study^21^ conducted in southwest China based on 10-year follow-up data, 5625 participants (2563 males and 3062 females) were included in the final analysis. Heavy smokers had a higher risk of hypertension than non-smokers (HR=1.50; 95% CI: 1.05–2.16).

One explanation for the link between smoking and hypertension is that cigarette smoking leads to endothelial dysfunction, a key factor in the development of hypertension. Endothelial dysfunction refers to impairment of the inner lining of blood vessels, leading to reduced availability of nitric oxide and increased vasoconstriction. This can result in increased peripheral resistance and elevated BP levels^22^.

Notably, the exact mechanisms underlying BP reduction after smoking cessation are not yet fully understood. However, the restoration of endothelial function, decreased oxidative stress, and reduced sympathetic nervous system activity may contribute to improvements in BP control^22^. In this regard, a reduction in HR could serve as a marker of decreased sympathetic activity^23^. Additionally, based on the association between CO and HR, our data suggest that HR influences the decrease in BP through central mechanisms. Further studies are required to identify factors involved in BP reduction. In addition, smoking cessation has been shown to have beneficial effects on BP control. These findings underscore the importance of smoking cessation for mitigating the effects of smoking on BP.

Finally, it is crucial to address the safety of smoking cessation drugs, specifically varenicline and bupropion, because of the past uncertainties surrounding the cardiovascular safety of these drugs^24^. Our study showed that these drugs did not lead to any further increases in BP in smokers who did not quit smoking, reinforcing previous evidence regarding the safety of these drugs with respect to hemodynamic parameters, as previously demonstrated^25^. These findings support the use of varenicline and bupropion for smoking cessation.

Another aspect to be discussed is the significant difference in weight gain among participants who quit smoking. This study showed that among hypertensive smokers who quit, weight gain was 1.5 kg at Week 12, albeit with BP reduction. Despite the benefits of smoking cessation, data indicate that weight gain is frequent among patients who stop smoking^26^. Over 9 years of follow-up, weight gain was approximately 3–4 kg higher in women who quit smoking than in those who continued smoking or never smoked at all^27^. Despite moderate differences in BP, smoking cessation was associated with an increased incidence of hypertension. In a previous study with over 800 smokers^28^ who underwent smoking cessation treatment at a specialized smoking cessation center between 2008 and 2012, weight gain occurred in 75% of patients who quit smoking after 52 weeks of follow-up. Among those who gained weight, average gain was 6.7 kg for women and 3.8 kg for men, 1 year after smoking cessation. No increase in BP was observed in this population.

When smoking cessation is associated with weight gain of >10%, there is concern that weight gain-related metabolic disorders could counterbalance the advantages gained with smoking cessation^29^. Studies that assess the impact of weight gain in the general population correlate with an increased risk of mortality for every 5-unit increase in BMI^29^. Berhe et al.^30^ studied a representative sample of 16663 Australian patients aged >18 years who quit smoking and gained weight, and followed them in a cohort study from 2006 to 2014. Smoking status, anthropometric data, risk of cardiovascular disease, type 2 diabetes, cancer, chronic obstructive pulmonary disease, and overall mortality were evaluated. That study concluded that smoking cessation was associated with weight gain, ranging from 1 to 10 kg or 0.1 to 0.2 increment in BMI during the evaluation period of six years, but the benefits of smoking cessation outweighed the risks and reduced mortality in the general population. No increase in the risk of chronic diseases was observed, regardless of weight gain or BMI change after smoking cessation. In conclusion, those authors stated that the benefits of smoking cessation outweighed the risks associated with weight gain.

The consensus is that smoking cessation, which is associated with weight gain, results in undeniable survival benefits, especially BP reduction in hypertensive smokers. However, excessive weight gain comes with other limitations that translate into a loss of quality of life and may act as a discouraging factor for smoking cessation treatment. Physicians should encourage their patients to quit smoking using pharmacological strategies and promote physical activities that minimize weight gain and positively impact life expectancy and quality of life.

Collectively, these studies demonstrated that smoking cessation has a positive impact on BP control in hypertensive smokers. Smoking cessation is crucial to manage hypertension and reduce the risk of cardiovascular complications. Hypertension is a condition characterized by chronically elevated BP levels, significantly increasing the risk of cardiovascular diseases such as heart attacks and strokes^31^. The combination of hypertension and smoking further increases the risk of myocardial infarction, as demonstrated in the INTERHEART case-control study^1^. In addition to reducing cardiovascular morbidity/mortality and overall mortality^2^, smoking cessation also plays a vital role in improving BP control in hypertensive smokers.

### Limitations

First, analysis involved only the subgroup of smokers with hypertension, resulting in a smaller sample size (113 smokers) when compared to the 361 smokers randomized in GENTSMOKING. This may have impacted the analysis of CO and HR associations; therefore, a causal relationship between these variables could not be established. Another limitation was that no long-term follow-up was conducted at this time, as well the lack of information about physical activity in the study population, as this parameter affects both heart pressure and weight gain. Laboratory parameters like metabolic function and renal function were not explored in this analysis. Finally, there may be additional residual confounding factors due to non-controlled adjustments that were not assessed in this study, such as lifestyle changes and endothelial function.

## CONCLUSIONS

Smoking cessation led to a significant reduction in BP in hypertensive participants, especially in hypertensive smokers more distant from the control goal. The beneficial impact of BP control in hypertensive smokers translates to a reduced risk of cardiovascular complications associated with hypertension. These findings, along with the additional evidence presented, should be emphasized in hypertension treatment guidelines, highlighting the role of smoking cessation in improving BP control and potentially reducing the need for additional antihypertensive drugs.

## Data Availability

The data supporting this research are available from the authors on reasonable request.
